# Effects of the combination of methylprednisolone with aminoguanidine on functional recovery in rats following spinal cord injury

**DOI:** 10.3892/etm.2014.1613

**Published:** 2014-03-10

**Authors:** ZONGSHU LI, JUAN DU, HONGXIA SUN, JING MANG, JINTING HE, JIAOQI WANG, HONGYU LIU, ZHONGXIN XU

**Affiliations:** 1 Department of Neurology, China-Japan Union Hospital of Jilin University, Changchun, Jilin 130033, P.R. China; 2 Department of Neurology, Jilin Province People’s Hospital, Changchun, Jilin 130021, P.R. China; 3 Department of Rheumatology and Immunology, China-Japan Union Hospital of Jilin University, Changchun, Jilin 130033, P.R. China

**Keywords:** spinal cord injury, methylprednisolone, aminoguanidine, functional recovery

## Abstract

Methylprednisolone (MP), a synthetic glucocorticoid, has been widely used as a standard therapeutic agent for the treatment of spinal cord injury (SCI). The combination of MP and other pharmacological agents aimed at enhancing functional recovery is desirable as the beneficial effects of MP are controversial, due to a variety of side-effects. Aminoguanidine (AG), a small water-soluble compound, is potentially useful in the treatment of acute SCI. The aim of the present study was to determine the effects of MP and AG, administered in combination, following SCI in adult rats. In rats with SCI, the combination therapy group treated with AG (75 mg/kg) and MP (0.75 mg/kg) exhibited significantly reduced levels of cytokine expression and cell apoptosis compared with those in the control group. In addition, the data demonstrated that the combination therapy significantly enhanced the recovery of limb function. These data clearly suggest that treatment with a combination of MP and AG represents a promising strategy of clinically applicable pharmacological therapy for the rapid initiation of neuroprotection following SCI.

## Introduction

The pathophysiology of traumatic spinal cord injury (SCI) is thought to divide into two stages (1). As the primary insult, the direct mechanical damage cannot be therapeutically influenced. However, the secondary damage, including electrolyte abnormalities, free radical formation, edema, vascular ischemia, posttraumatic inflammatory reaction, apoptosis and other processes, may be targeted with various therapeutic interventions. It has been shown that inflammatory processes play an important role in post-SCI secondary injury (2–4). Therefore, it is important to develop a therapy that reduces the evolution of the secondary damage in SCI.

The spinal cord is a glucocorticoid-responsive tissue and it contains substantial amounts of receptors for adrenocortical steroids (5,6). It has been demonstrated that glucocorticoid drugs enhance functional recovery and induce regenerative responses following SCI in humans and experimental animals (7,8). Methylprednisolone (MP) is a synthetic glucocorticoid and the only therapeutic agent approved by the Food and Drug Administration for reducing the extent of the post-traumatic inflammatory reaction following acute SCI (9,10). Although the application of MP after SCI is associated with a wide array of anti-inflammatory effects, including anti-lipid peroxidation (11,12) and attenuation of the formation of deleterious prostanoids (prostaglandin F2α and thromboxane A2) (13), the long-term administration of this therapeutic steroid results in a variety of side-effects, such as downregulation of the expression of several inflammatory genes and an inhibitory effect on the proliferation of endogenous neural progenitor cells following SCI (14).

Aminoguanidine (AG), a small water-soluble compound, has been widely used for the prevention of the chronic tissue complications of diabetes mellitus in humans (15). Previous studies have shown that AG reduces the extent of brain edema in animal models of surgical brain injury (16), stroke (17) and post-traumatic brain injury (18). Notably, Pearse *et al* demonstrated that AG improved the motor functions of injured spinal cords in rats and may have a potential role in the treatment of acute SCI (19). However, high doses of AG lead to nonspecific and potentially toxic effects (20), which limits its usefulness clinically. Treating a single target with a low dose of therapeutic agent is unlikely to achieve complete inhibition of the inflammation due to the complexity and redundancy of the inflammatory response associated with SCI. It has been shown that the strategy of targeting multiple proinflammatory pathways may be more effective than targeting a single effector molecule (21,22). To the best of our knowledge, the therapeutic effects of the simultaneous administration of AG and MP have not previously been evaluated. Thus, to determine whether MP and AG act synergistically, SCI was induced in rats and the effects of MP and AG were determined in the present study.

## Materials and methods

### Experimental animal

Sixty Wistar adult female rats (200–240 g) were obtained from Jilin University (Changchun, China). All animals were enclosed in ventilated, humidity- (50–60%) and temperature-controlled (22±1°C) rooms with a 12/12-h light/dark cycle for approximately two weeks. The animals were housed on sawdust and received food pellets and water *ad libitum*. All animal procedures were performed in accordance with the Guide for the Care and Use of Laboratory Animals of the National Institutes of Health (1996) and were approved by the Jilin University Committee on Animal Research.

### Surgical procedure of SCI

The rats were anesthetized with a cocktail of 40 mg/kg ketamine, 4 mg/kg xylazine and 0.9 mg/kg acepromazine administered by intraperitoneal (IP) injection. A dorsal incision was made to expose the T10 vertebra and a laminectomy was performed, leaving the spinal segment exposed. Following exposure of the T10 segment by laminectomy, the animals received a moderate contusion using a New York University impactor (W.M. Keck Center for Collaborative Neuroscience, Rutgers the State University of New Jersey, Piscataway, NJ, USA) that provides a contusion of 12.5 g.cm as previously described (23). Following the surgery, 10 ml 0.9% sodium chloride and 30 mg/kg sulfadiazine and trimethoprim were injected subcutaneously. Access to food was facilitated by placing softened food pellets directly in the bottom of each cage. The state of hydration and gastrointestinal function were monitored daily. The rats were weighed daily for the first seven days postsurgery and then weighed weekly. Post-surgical care included the manual expression of bladders twice a day until bladder function returned, as well as injections of sulfadiazine and trimethoprim twice a day for up to one week.

### Experimental groups

The rats were randomly allocated into the following groups: Group 1: Sham surgery group, the animals were subjected to identical surgical procedures without impaction; group 2: Control group, the rats received an IP injection of the carrier solution (5 ml/kg of 5% dimethylsulfoxide in 0.9% normal saline) following SCI; group 3: MP group, MP (0.75 mg/kg, IP) was administered at 1 and 4 h after SCI according to the methods of Messina *et al* (22); group 4: AG group, AG (75 mg/kg, IP) was administered at 1 and 4 h after SCI; and group 5: AG and MP group, AG (75 mg/kg, IP) and MP (0.75 mg/kg, IP) were administered at 1 and 4 h after SCI. One rat of each group was used in each of the following experiments.

### Determination of spinal cord water content

The spinal cords collected 24 h after treatment. Spinal cord edema was evaluated by determining the water content of the spinal cord as previously described with minor revision (21). In brief, the injured spinal cords for all groups were dried for 48 h at 80°C for determination of the dry weight. The values for the water content in the spinal cord tissues were obtained based on the following calculation: Hemispheric water content (%) = (wet weight - dry weight)/wet weight × 100.

### Myeloperoxidase (MPO) activity

The levels of MPO activity, an indicator of polymorphonuclear leukocyte accumulation, were determined in the spinal cord tissues according to the methods of a previous study (24) at 24 h after SCI. MPO activity was defined as the quantity of enzyme required to degrade 1 μmol of peroxide per min at 37°C and was expressed in U/g of wet tissue.

### Behavioral assessments

Behavioral assessments were determined using the Basso, Beattie, and Bresnahan (BBB) score and grid-walking test. Gross BBB locomotor recovery following contusive SCI was scored in an open field according to the locomotor rating scale of 0 (complete paralysis) to 21 (normal locomotion) (25). BBB testing was performed at 24 h prior to SCI, 24 h and 3 days post-injury, and once weekly thereafter up to eight weeks post-injury. Each rat was observed for 4 min by three blinded investigators. To assess the locomotion in all groups, the ability of rats to walk on an irregularly horizontal wire grid was determined as described by a previous study (26). The rats were allowed to walk on the grid weekly and tested at eight weeks after the contusive SCI. Each rat was allowed to walk around freely for 4 min. If a hind paw protruded entirely through the grid, with all toes and the heel extended below the wire surface, it was counted as a misstep. Furthermore, the total number of steps taken with the hindlimb of the same side was also counted. The results are shown as a percentage of missteps.

### Measurement of tumor necrosis factor-α (TNF-α) and interleukin-1β (IL-1β) levels following SCI

To evaluate the TNF-α and IL-1β tissue levels, sections of the spinal cord tissues, collected at 24 h after SCI, were homogenized as previously described (21) in phosphate-buffered saline containing 2 mmol/l phenylmethylsulfonyl fluoride (Sigma Chemical Co., Milan, Italy). The assay was performed using a commercial colorimetric kit (rat TNF-α commercial colorimetric kit and rat IL-1β commercial colorimetric kit; Calbiochem-Novabiochem Corporation, San Diego, CA, USA) according to the manufacturer’s instructions. All detections were performed in duplicate serial dilutions.

### Western blot analysis

Western blot analysis was performed to investigate the expression levels of the Bcl-2-associated X (Bax) and B-cell lymphoma 2 (Bcl-2) proteins in an extract from the injured spinal cord at 24 h after SCI. Following sacrifice under deep anesthesia by transcardial saline infusion, the experimental rat spinal cord tissue (1.5-mm long, centered at the injury site) was quickly removed and homogenized by sonication in radioimmunoprecipitation assay lysis buffer. The samples were centrifuged at 12,000 × g for 1 h. The protein concentration of the soluble materials was determined by the Coomassie Brilliant Blue G-250 dye-binding method (Thermo Fisher, Rockford, IL, USA). The protein lysates (15 μg per lane for each sample) were fractioned by 10% SDS-PAGE, followed by transfer to nitrocellulose membranes (Santa Cruz Biotechnology, Inc., Santa Cruz, CA, USA). The membranes were blocked in blocking buffer (5% nonfat dairy milk dissolved in Tris-buffered saline with Tween 20 and PBS with Tween 20) overnight at 4°C. The blots were then incubated with anti-Bax and anti-Bcl-2 rabbit polyclonal antibodies (dilution 1:500; Santa Cruz Biotechnology, Inc.) for 2 h. The Bax and Bcl-2 protein bands on these immunoblots were visualized using enhanced chemiluminescence (ECL) western blotting kit (Santa Cruz Biotechnology, Inc.). The Bax and Bcl-2 protein bands and GAPDH bands were scanned using the ChemiImager 5500 system with the corresponding software, version 2.03 (Informer Technologies, Inc., Dallas, TX, USA), and the integrated density values were calculated using FluorChem software, version 2.0 (Informer Technologies, Inc.) and normalized with those of GAPDH.

### Statistics analysis

The statistical package SPSS software, version 19.0 (SPSS, Inc., Chicago, IL, USA) was used for all analyses. One-way analysis of variance followed by Bonferroni’s post hoc test were utilized to determine the significant differences among multiple groups. All values are expressed as the mean ± standard deviation. In general, P<0.05 was considered to indicate a statistically significant difference.

## Results

### Effect of the combination therapy on spinal cord water content

**Figure 1:**
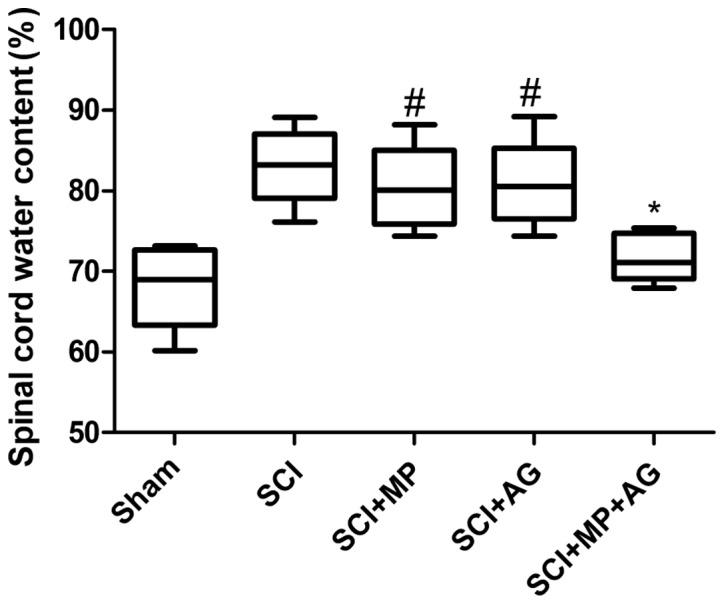
Changes in the percentage water content in the SCI areas in all groups at 24 h after SCI. The combination therapy with AG (75 mg/kg) and MP (0.75 mg/kg) significantly ameliorated the water content of the injured spinal cord compared with the effect of a single treatment (AG or MP). ^*^P<0.05 versus the control (SCI) group, ^#^P<0.05 versus the sham group. SCI, spinal cord injury; MP, methylprednisolone; AG, aminoguanidine.

In the present study, the effect of combination therapy with AG (75 mg/kg) and MP (0.75 mg/kg) on the spinal cord water content at 24 h after SCI was investigated. As shown in Fig. 1, the combination therapy had significant anti-edematous activity compared with that observed in the control group (SCI group), whereas in the single treatment groups (the MP and AG groups) the levels of cerebral edema did not significantly change compared with those of the control group at 24 h after SCI. However, the levels of cerebral edema in the single treatment groups were significantly increased compared with those of the sham group. These data showed that the combination therapy with AG and MP significantly ameliorated the increased water content of the injured spinal cords.

### Effect of the combination therapy on neutrophil infiltration

**Figure 2:**
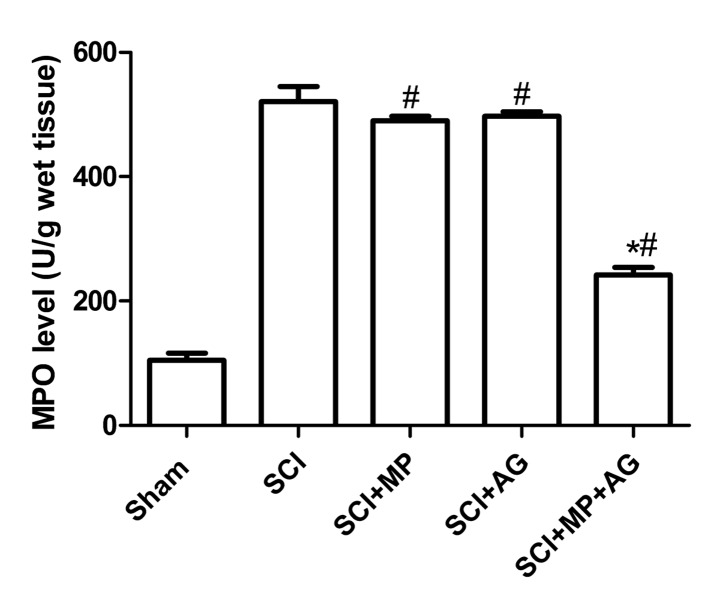
Effects of the combination therapy on the levels of MPO activity. Following the injury, the levels of MPO activity in the spinal cords from SCI rats were significantly increased at 24 h after the damage in comparison with those of the sham surgery group. Treatment with a combination of AG and MP significantly reduced the SCI-induced increase in MPO activity. A single treatment (MP or AG) did not reduce the levels of neutrophil infiltration in the injured spinal cords. ^*^P<0.05 versus the control (SCI) group, ^#^P<0.05 versus the sham group. MPO, myeloperoxidase; SCI, spinal cord injury; MP, methylprednisolone; AG, aminoguanidine.

The effect of combination therapy with AG (75 mg/kg) and MP (0.75 mg/kg) on neutrophil infiltration was investigated by measuring the tissue levels of MPO activity. MPO activity was significantly elevated in the spinal cord at 24 h after injury in the rats subjected to SCI when compared with those of the rats in the sham surgery group (Fig. 2). The levels of MPO activity were significantly reduced by the combination therapy with AG (75 mg/kg) and MP (0.75 mg/kg) compared with those of the rats in the control group (Fig. 2). However, administering either of the compounds as a single treatment did not reduce the levels of neutrophil infiltration in the injured spinal cord compared with those of the rats in the control group (Fig. 2).

### Combination treatment with MP and AG results in functional recovery following SCI

**Figure 3:**
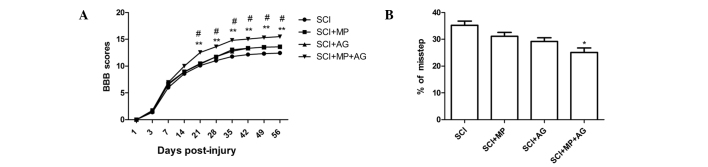
Combination treatment with MP and AG improved functional recovery following SCI. (A) BBB tests were performed at various time periods following contusive SCI. ^**^P<0.01 as compared with the control (SCI) group, ^#^P<0.05 as compared with the AG or MP groups. (B) The grid walking test showed that the percentage of missteps in the combination-treated group was significantly lower than that in the control group (^*^P<0.05). BBB, Basso, Beattie, and Bresnahan; SCI, spinal cord injury; MP, methylprednisolone; AG, aminoguanidine.

To determine whether the AG and MP combination treatment-mediated tissue protection and repair also had an effect on functional recovery, the BBB locomotor test was performed at 1 day, 3 days and weekly up to eight weeks after SCI (Fig. 3A). At 1 day after SCI, BBB score of all rats was regarded as 0. In the following days, the locomotor performance substantially improved and reached a relative plateau at the third week. A minor but not statistically significant increase of the BBB scores was observed in the AG and MP groups from the fifth week after SCI. The scores in the AG and MP combination treatment group were consistently higher than those in the other groups and the differences between BBB scores of the AG and MP combination group and those of the other three groups were statistically significant starting from the third week and continuing until the eight week. The rats subjected to sham surgery all achieved maximal scores in the BBB test (data not shown). Results from the grid walking test also showed that the percentage of missteps of hind paws was markedly reduced in the combination-treated rats compared with that in the other groups (Fig. 3B).

### Effect of the combination therapy on the expression levels of TNF-α and IL-1β following SCI

**Figure 4:**
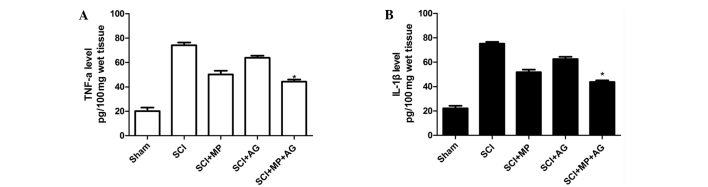
Effects of the combination therapy on the TNF-α and IL-1β levels of the injured spinal cords. Substantial increasse in the levels of TNF-α and IL-1β production were identified in the spinal cord tissues from the SCI rats at 24 h after the SCI. The combination therapy with AG and MP significantly reduced the TNF-α and IL-1β levels.^*^P<0.05 versus the control (SCI) group. TNF-α; tumor necrosis factor-α; SCI, spinal cord injury; MP, methylprednisolone; AG, aminoguanidine; IL-β, interleukin-1β.

To test whether the combination therapy with MP and AG modulated the inflammatory process through regulation of the secretion of proinflammatory cytokines, TNF-α and IL-1β levels in the spinal cord tissues were analyzed. Substantial increases in the levels of TNF-α and IL-1β production were identified in the spinal cord tissue samples collected from SCI rats at 24 h after SCI (Fig. 4). The combination therapy significantly reduced the spinal cord levels of TNF-α and IL-1β compared with those of the control group (Fig. 4). Furthermore, the MP treatment group exhibited markedly reduced spinal cord levels of TNF-α and IL-1β compared with those of the control group (SCI group), whereas the AG treatment group did not show clearly reduced TNF-α and IL-1β levels compared with those of the control group (SCI group).

### Western blot analysis of Bax and Bcl-2

**Figure 5:**
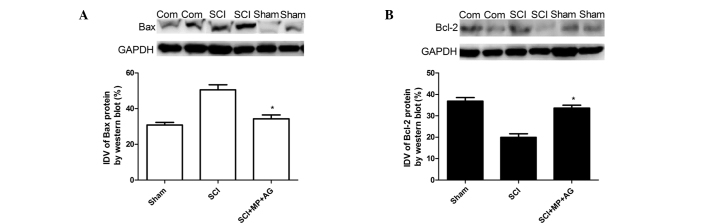
Effects of the combination therapy on (A) Bax and (B) Bcl-2 expression levels in the injured spinal cord site at 24 h after the SCI. ^*^P<0.05 versus the control (SCI) group. Com, combination; SCI, spinal cord injury; Bax, Bcl-2-associated X protein; IDV, integrated density values; MP, methylprednisolone; AG, aminoguanidine; Bcl-2, B-cell lymphoma 2.

At 24 h after SCI, the appearance of Bax in the spinal cord homogenates was investigated by western blot analysis. The Bax expression levels were appreciably increased in the spinal cords from the rats subjected to SCI, compared with those in the spinal cord from the sham rats (Fig. 5). However, the combination therapy with MP and AG significantly reduced Bax expression levels compared with those in the control group (Fig. 5). Furthermore, whole extracts from the spinal cord of each rat were also analyzed to detect Bcl-2 expression levels. Combination treatment of the rats with MP and AG significantly increased the SCI induced inhibition of Bcl-2 expression (Fig. 5). These results indicate that combination therapy with MP and AG inhibits apoptosis following SCI.

## Discussion

The present study provides convincing evidence that the combination of MP with AG significantly reduced the levels of spinal cord edema and improved the damaged motor function caused by SCI in rats, whereas a single treatment did not significantly improve them.

Although the two compounds have each been extensively studied, to our knowledge, this study is the first to show an enhanced neurological outcome from combining a clinically applied therapy, MP, with AG following SCI. The enhanced viability and regenerative capacity of neurons and functional recovery supported by MP and AG combination treatment has practical and conceptual implications due to the proinflammatory effect on neurons *in vivo* following experimental SCI (19,27,28).

Traumatic SCI results in severe inflammation, the release of free oxygen radicals, a reduction of neural regeneration and glial scar formation, all of which are detrimental to neural function recovery (29). It is unrealistic to expect to achieve disease remission by blocking a single early mediator in the inflammatory cascade as a large number of inflammatory mediators are involved in the secondary injury processes following SCI. Therefore, the observed combination therapy is of potential therapeutic interest and suggests that strategies targeting multiple proinflammatory pathways may be more effective than those targeting a single effector molecule. Yin *et al* (30) showed that rolipram and MP combination treatment promoted significant neuroprotection in rats through reduced motor neuron death, a minimized lesion cavity and increased regeneration of lesioned corticospinal tract axons beyond the lesion site following SCI. Genovese *et al* (31) demonstrated that treatment of mice with a combination of etanercept and dexamethasone (DEX) significantly reduced the SCI-induced spinal cord changes and also improved the motor function compared with the effects of etanercept or DEX treatment used alone. Xu *et al* (21) showed that combination therapy with AG and DEX significantly exerted an important beneficial anti-inflammatory effect by blocking the possible progression of SCI in rats. These studies demonstrated that combination therapy significantly ameliorates functional recovery following SCI compared with the effect of single drug treatment. The present study showed that the treatment of SCI rats with MP and AG, when administered as a combination therapy but not as a single treatment, significantly reduced the levels of neutrophil infiltration (MPO activity), cytokine expression (TNF-α and IL-1β) and apoptosis (Bax and Bcl-2 expression) compared with those of untreated rats, and it demonstrated that the combination therapy significantly improved the recovery of limb function. These results further confirmed that strategies targeting multiple proinflammatory pathways may be more effective than those using a single drug molecule.

Spinal cord trauma initiates a sequence of events that lead to secondary neuronal cell damage. An inflammatory response develops within hours after injury and is characterized by the infiltration of neutrophils and the activation of microglia (32). Reactive microglia have been considered to be at the center of the injury cascade (33). Through releasing molecules, including TNF-α, IL-1β, reactive free radicals and nitric oxide, microglia encourage early post-injury necrotic cell death, remote cell apoptosis, tissue edema and axonal degeneration (34,35). Thus, methods of modulating microglia activation via the inhibition of cell cytokines to improve recovery following SCI are sought. The present study clearly demonstrates significant increases in the levels of TNF-α and IL-1β in rats with SCI. Combination therapy with MP and AG significantly reduced the levels of cytokine expression (TNF-α and IL-1β), which indicated that combination therapy with MP and AG may be effective treatment method for SCI.

In conclusion, the data imply that strategies targeting multiple proinflammatory pathways may be more effective than those targeting a single effector molecule. The combination therapy with AG and MP was shown to significantly exert an important, beneficial anti-inflammatory effect by blocking the possible progression of SCI; however, the detailed molecular mechanism by which the combination therapy with AG and MP treats SCI is unclear and requires investigation in further studies.
